# Modern Amalgam Restorations

**Published:** 1917-09

**Authors:** J. V. Conzett

**Affiliations:** Dubuque, Iowa


### MODERN AMALGAM RESTORATIONS

BY J. V. CONZETT, D.D.S., DUBUQUE, IOWA

[Read before the National Dental Association at its Twentieth Annual Session, Louisville, Ky., July 25-28, 1916.]

One of our authorities on amalgam has recently said that 75 per cent. of the restorations of the human teeth were made with amalgam. Whether that is exactly true or not every observant operator knows that at least the majority of the fillings that he sees are amalgam. And the very large majority are made with absolutely no regard to the science of cavity preparation, or of the proper manipulation of the material, and the result is anything but useful or esthetic. This being true, it has seemed well to the promoters of this section to present the modern method of amalgam restoration and to emphasize the fact that fillings can be made with the material that are useful in the highest degree, and, aside from the color, are esthetic, in that they can be made to represent the form and size of the injured tooth.

The great reason for the larger use of amalgam over any other material is the supposed ease of its insertion and its cheapness. It is *not* easy to make a good amalgam filling. It requires as much care and skill as does a foil filling or one made with the inlay, but its *apparent* ease of insertion appeals to the careless or indifferent operator, and the reason we see so many poor fillings made with this much-abused material is for this reason. If any of the men who are laboring under this delusion will make a few fillings and have them tested for strength, adaptation and permanence of form under stress, he will rapidly come to the conclusion that it is the hardest material we possess to perfectly and permanently adapt to the walls of a cavity. This is particularly true of the old alloys which, by reason of their unscientific methods of manufacture and their faulty formulas, were lamentably weak under the stress of mastication, and continued to grow weaker with age, so that a filling made with some of them would change its shape to such a degree that it absolutely failed to prevent the ingress of moisture between filling and tooth, with a consequent recurrence of decay. So much was this true that when ten years ago the writer said that he found decay under the majority of amalgam fillings that he removed, one of the men who discussed the paper and one of the ablest men in the profession, said that you would *always* find decay under an old amalgam filling. That statement was not absolutely true at that time and certainly is not today, but the remark showed the poor opinion that the leading men of the profession had of the material.

But whatever may have been the fault of the material of the past, whatever may have been wrong in the manipulation and insertion of amalgam twenty years ago, the fact remains that today, thanks to the genius and labor of Dr. G. V. Black, we have a material and a method that, if carefully followed, will result in a filling that will permanently and usefully restore broken-down tooth tissue, and the more they are broken the more is the material indicated.

In the making of a good amalgam filling there must be a proper cavity preparation, a good alloy and the proper manipulation thereof, the proper insertion of the amalgam into the cavity and a proper trimming and finishing of the built-in filling, and in this paper we will attempt to follow in sequence the making of an amalgam filling in harmony with modern ideas upon the subject as they have been developed by the men that have studied the subject along the lines of modern scientific investigation.

First there must be a proper cavity preparation.

The science of cavity preparation as developed by Dr. Black is fundamental and pertains to any and all cavities irrespective of the material with which the cavity is to be filled, and only such changes are made in the form of the cavity in using different materials as the exigencies of the material may demand. Fundamentally all cavities are the same, and only such changes are made as are necessary to employ the material contemplated. For instance, in the making of an inlay it is obvious that there can be no under cut of any kind while in the making of a cavity for a gold filling a slight retention in this regard is permissible, and in the making of a porcelain inlay the beveling of the cavo-surface angle is forbidden because of the weakness of the restoring material, while in the making of any of the metal fillings or inlays the beveling of the cavo-surface angle is one of the most important considerations and is demanded by all careful operators.

Therefore, all that has been written in the past in regard to cavity preparation will apply to the making of an amalgam filling, but to properly develop our paper we will, at the risk of repetition, emphasize the salient points of Dr. Black's system of cavity preparation. He said:

*First.* Obtain the outline form.
*Second.* The resistance form.
*Third.* The retentive form.
*Fourth.* The convenience form.
*Fifth.* Remove any remaining carious dentine.
*Sixth.* Finish the enamel wall.
*Seventh.* Make the toilet of the cavity.

The outline form contemplates extension for prevention and the esthetic form. Extension for prevention means the carrying of all the margins of the cavity into areas that are comparatively immune to decay. It will not be possible in the time allotted to this paper to enter into a discussion of the doctrine of extension for prevention; suffice it to say that all cavities, no matter how small may be the initial decay, should be carried far enough that the margins of the finished filling will lie in immune territory.

That portion of the tooth is that which is kept clean by the excursion of food in mastication and by the movement of the tongue and lips, also all smooth occlusal surfaces and the substance lying under the gum. All cavities should be cut, therefore, far enough buccally and lingually that the lines of the cavity will be clear of the embrasures and will not be in contact with either tooth substance or filling in the approximating tooth, far enough gingivally that the margin will be covered by the gum tissue and far enough occlusally that the finished margin will be in contact with a smooth surface—that is that all fissures will be cut out to their extent—for if this is not done and the margin of the cavity is in a fissure or pit, the resulting rough margin will invite the retention of food debris and a recurrence of decay.

The esthetic form is that form that is given to a cavity that will in the highest degree conserve the appearance of the tooth. In an amalgam filling we do not look very much for esthetics, for we commonly expect to place the material only in those portions that will not be within the range of vision, and rightly so, or owing to its color an amalgam filling should never be placed anterior to the distal surface of a second bicuspid, and the writer rarely uses it in front of the distal surface of the first molar, nevertheless the beauty of the restoration should never be lost sight of. We should train ourselves to be artists and not merely artisans, and our ideal should always be the most beautiful operation consistent with the condition under consideration. We should always have a high ideal, and though we will rarely reach the goal, the ever aspiring for it will make us continually advance in our operative endeavors. So an amalgam filling should be made as beautiful as the nature of the material will permit. In the making of a cavity, therefore, the lines of the cavity should conform to the rules of art as closely as possible, and all external angles should be made with a slight curve, for nature abhors an angle. The shape of the tooth to be restored should be carefully studied and the shape reproduced as closely as possible. The highest art is to hide art and reproduce nature as closely as possible, and while this is not possible as it relates to color in amalgam restoration, it is relevant when we consider the size and shape of the tooth. All restorations should approximate the natural size, shape and position in the arch of the natural tooth as closely as it is possible for the operator to do so.

The resistance form is that which is given to the cavity that will in the highest degree resist the thrust forces that will come upon the finished filling. The best form we can devise for this purpose is that of a box. Flat seats and parallel walls are demanded in this form of cavity preparation. It would not seem to be necessary to emphasize this point to any one with the slightest knowledge of mechanics, but habit is strong, and the prejudice of long-continued operations along another line make many operators resist this form of preparation and insist that a rounded base is good enough. Nothing is good enough that is not perfect, and if another method is born that is better than the one now advocated, we will be the first to espouse it. A flat base gives the most perfect foundation for any structure, and its shape causes it to divide the load over its entire surface, while a rounded base will cause certain portions of the base to bear the entire load at one time and another part of the load at another time. Furthermore, a structure built upon a rounded base will have a tendency to rock under stress to the great detriment of the stability of the entire filling, and even the tooth itself is laid under such a strain that it will be liable to break. Therefore we insist upon a flat seat, and walls that are as parallel with one another as it is possible for us to obtain. It will not be possible for us to particularize in a paper of this nature; we will have to generalize and merely give fundamentals, but we will follow the reading of the paper with slides showing the different preparations advocated, and this will convey to the mind the principles that we desire to show better than the spoken word can do.

The retentive form is that which we give to a cavity that will resist the pull forces that will have a tendency to lift the filling out of the cavity. This form is obtained by parallel and slightly undercut walls. The parallel wall is amply sufficient if enough force is obtained in condensing the filling, which can only be done if the amalgam is fairly dry and the mallet used in condensing the material for the dentine is elastic and under sufficient stress will give a little, and upon the removal of the stress will have a tendency to resume its normal condition; therefore, if the filling material is forced into the cavity with sufficient force to cause the dentine to give, its elasticity will cause it to hug the filling so tightly that it could only be displaced by a force that would be sufficient to break the tooth.

The convenience form is that which we give to a cavity that enables us to perfectly adapt the filling material to the walls of the tooth, for no matter how perfectly our cavity may be prepared, if the filling material is not so introduced that it will hermetically seal the cavity, the filling will be a failure. It is not possible for anyone to tell anyone else just how to cut every cavity that he is to fill, for a cavity that would be difficult for me to fill might be easy for you, or conversely, one that I might be able to perfectly fill might be impossible for you. The point is to always cut a cavity sufficiently wide and make it accessible enough for you to perfectly fill it. And that holds good of a filling made with any material or method. Someone that had been watching Dr. Black operate in his office was asked if he had filled any difficult cavities, and he said, "No; all of the cavities that Dr. Black filled while I was there were easy to fill, for he made them so." Amalgam is one of the most difficult of materials to perfectly adapt to a cavity wall, and in making a cavity for its reception we should always bear this in mind and make the entrance of the cavity of sufficient proportions that we will be able to easily adapt the material to its walls.

Remove any remaining carious dentine. The walls have been carried out to sound, strong tissue, the base has been squared and the walls properly prepared, and in doing this we have removed most of the carious dentine, but this has not been our first thought, so we now carefully examine the remaining tissue and test it for carious spots, for it must not be forgotten that the removal of all decayed dentine is absolutely imperative, and if not done, invites failure as sure as the filling is so made. One of the great reasons that we do find decay so frequently under amalgam fillings is the fact that men are not as careful to remove all of the decay in making a cavity for the reception of amalgam as they are when they are making one for the reception of gold. They seem to think that gold requires a more careful preparation than does a cavity for the insertion of amalgam, but, if anything, the converse is true. Certain it is that all decay should be carefully removed from a cavity, no matter what the material is that is to make the filling.

Prepare the enamel walls. The beveling of the cavo-surface angle must be as carefully done as for a gold filling and for the same reasons. The only difference is that in the practice of the writer the bevel is made longer for an amalgam filling than for a gold one, for the reason that amalgam has not the edge strength of gold and a larger amount of the material as it bulks against the enamel walls makes for a greater strength. We use about the same bevel for an amalgam filling as we do for a gold inlay and use the same angle, which is one of about 12½ centigrades. This angle makes a margin that flows into the cavity and makes a strong resistant margin. We insist upon the beveling of the cavo-surface angle for the protection of the enamel margin, for we know that a margin that is not beveled will contain a multitude of short enamel rods that will have little or no strength and will fall out under the stress of mastication and leave a vulnerable spot for the beginnings of a recurrence of decay.

Make the toilet of the cavity. If the rubber dam has been in place, and this is the ideal, it will only be necessary to cleanse the cavity of the chips and debris made by the preparation of the cavity and proceed with the filling. In case the dam has not been placed, and in the practice of the large majority of operators it is not for amalgam, then the cavity must be thoroughly washed out with warm water and then either the dam applied or some means adopted that will insure a dry cavity until the completion of the filling. When the cavity is dried it should be washed out with alcohol or chloroform, dried again, and is then ready for the filling.

The next step is the selection of an alloy from which to make the filling, and in this day this is not a hard proposition, for the Black formula alloys are being placed upon the market by a number of firms, and a splendid product is the result. It is not the province of the writer to recommend the product of any firm; suffice it to say that he only insists upon using an alloy that is made according to the findings of Dr. Black and one that does not contain zinc. We are assured that zinc does no good in an amalgam and can and does do a great deal of harm. For instance, we are told by Dr. Black that the presence of zinc makes an amalgam that becomes increasingly weaker with age. And we know that by reason of the great difference in electrical potential between zinc and gold it is certainly contraindicated in a mouth that contains any gold, for the presence of gold in the same mouth with a zinc alloy will create an electric current that will cause a galvanic action between the different elements and the zinc will be dissolved out of the alloy, leaving it pitted and rough, to say nothing of the effect such action might have upon the teeth or the system. Dr. Clarence Grieves demonstrated the truth of this assertion some time ago in his experiments with zinc alloyed orthodontia appliances. There are several excellent silver-tin-copper alloys upon the market, and the writer uses one of several of these in his operations.

Having selected the alloy, it is necessary to obtain a pure mercury, for a contaminated article will introduce into the amalgam a sufficient amount of foreign material to break up the balance of the amalgam, and the result of the careful work of the manufacturer will have gone for naught.

Definite methods obtain definite results, so a definite amount of alloy and mercury used in each mix will always obtain the same result in the amalgamated mass. It is essential, then, to use some method of always having the same amounts of the two elements in every mix. This can be obtained by weighing the elements each time that a mix is to be made, or better still, the amounts of alloy and mercury are weighed out beforehand and the same placed in capsules ready against the time that they are to be used. In this way the minimum of time is consumed in getting ready for the mix, and if you use your assistant in making the mix, you are sure that she is always obtaining the same result.

There is a difference of opinion and a conflict of authorities in regard to the consistency of the mix. Some men demanding an amalgam that is so dry that it is almost impossible to get it into the cavity before it has set, and on the other extreme are the men who are advocating the sloppy mix. In the opinion of the writer both ends of the discussion are wrong. If the mix is too dry it is practically impossible to make a filling that is impervious to moisture, and if a sloppy mix is used, the sacrifice of the strength of the amalgam is too great.

It is true that a moisture-proof filling can more easily be made with a sloppy mix than with one that is stiffer, but you sacrifice strength to ease in doing so, and to the writer that is a questionable expedient. It is also true that with a sloppy mix you can not take advantage of the elasticity of the dentine in the condensation of your filling and thereby lose a great deal of retention and resistance to stress in the finished filling. I realize the fact that the advocates of the sloppy mix remove the surplus mercury as it comes to the surface in condensing the filling, but that is only an approximate method of obtaining results that we obtain by definite methods. The making of a filling in steel tubes is not analogous to the making of a filling in a tooth in the mouth, for the steel tube will have a rigidity that does not obtain in the tooth, and the advantage of the elasticity of the dentine is lost. In making a filling we use a mix that is fairly dry, not too much so, but one that will take the imprint of the markings of the thumb, as Dr. Black says. The first piece that is inserted may be a little softer than that and the following pieces be made a little dryer. These pieces are packed into the cavity with mallet pressure and are packed against the walls of the cavity with a stepping motion of the plugger exactly as in the case of a gold filling. In using the heavy condensing pressure of the hand mallet, we are forcing the amalgam into the cavity, exerting pressure upon the dentine, which in turn is compressed in proportion to the force used, and the elastic comeback after the condensation is finished helps hold the filling in place and makes a stronger and more resistant filling.

In making the mix a large mortar and pestle are advised and a definite amount of time in the trituration of the mass. And here again there is a conflict of authorities; Dr. Crandall and his followers advising a mix that is made with a short trituration, his point being that the mass is analogous to concrete with the granules of the alloy representing the rocks in the concrete and the mercury the cement. He claims, and demonstrates his point by experiment, that a mix so made is much stronger than one in which time is given for the complete amalgamation of the grains of alloy. On the other hand are those that demand a trituration of several minutes' duration, or until amalgamation of the entire mass has taken place, which makes a more easily worked mass, but one that does not have the resistance of the former.

Personally, I prefer the spatulation method, which gives a much quicker mix, a more homogeneous mass and the degree of amalgamation can be very easily determined. The method consists of taking the required amounts of the alloy and mercury and placing them in a long, narrow bottle in which they are shaken together with a violent agitation for a minute or two, when it will be found that the mercury has sufficiently adhered to the particles of alloy to enable the whole to be poured onto a piece of calendered paper and the mass spatulated with an ordinary putty knife. With four or five long movements of the spatula you will be surprised to find the mass quite thoroughly amalgamated, and with a little practice the suitable amalgamation can be easily obtained. This is the easiest and most efficient method of amalgamation that I have ever seen and is the one that is constantly used in my practice.

The nature of the amalgam is such that a good, impervious filling thoroughly adapted to the walls of the cavity can not be made unless there are four walls to the cavity. If one or more of the walls are missing, the mass will move out of the cavity in the direction of least resistance during the attempt to condense the filling. In order to prevent this we have to supply the missing wall or walls, and this is done with the matrix. There are a number of manufactured matrices upon the market, each one of which may have its particular merit, but the matrix that is made for the individual case is the better in the majority of cases. Sheet copper of 35 gauge is advised and may be obtained from the depots for this purpose. A suitable piece is cut and shaped and is then tied around the tooth with floss silk, the silk wrapped about the tooth and tied with an ordinary knot and then wrapped around again as many times as necessary to obtain the required shape and rigidity. Small wings are turned up at the corners of the copper at the gingival margin in order to engage the silk and prevent its slipping off of the metal and under the gingivae. When the matrix is in place a hole is cut at the contact point and a slit carried through the copper band to the occlusal, this to facilitate the tearing of the matrix after the filling is made and render the removal of the matrix easy. The hole through the matrix at the point of contact enables the operator to pack his amalgam directly against the approximating tooth and thus make the tightest possible contact point.

After the filling has hardened to a safe point the matrix is removed and the filling carved to form.

Instead of the copper a very good matrix may be made in the manner described by using the celluloid strips that may be obtained for use with the silicates. They are made in the same manner as those of the copper and are cut through in the same way, and have the advantage of imimparting a polish to the amalgam packed against it, and is easily torn apart when it is desired to remove the matrix.

The finishing of the filling is of the greatest importance and is most frequently neglected, and is one of the reasons for the horrible appearance of the majority of amalgam fillings. I am by no manner of means an advocate of sky prices for amalgam work. It is the poor man's filling material, and if it is not given to him for a price that he can pay, how in the world can he care for his teeth? The laborer is worthy of his hire, but the man with a big family and a small income can not pay five and ten dollars for a filling when there are almost numberless cavities to be filled, and if the price is not placed within his means, one of two things takes place; his family's teeth are neglected to the detriment of the health of the individual and the community, or he becomes a dead beat. I know that a good amalgam filling can be made that will serve the patient well and not make the fee so exorbitant that the patient will feel as though he had been robbed. Time is the great element in the fee question, and if we will develop the definite methods of making and filling, we can make one quickly, easily and at a profit to ourselves. But the filling must be shaped and properly finished if we are giving our patient honest service.

The contour should be properly made, the interproximal space restored and, almost most important of all, the occlusal aspect of the tooth properly reproduced. We have been told to finish the tooth anatomically, to restore the contact point and the marginal ridge, but it is only of late that sufficient emphasis has been placed upon the restoration of the sulçi, fissues and the occlusal planes. It is most important to do this not only from an utilitarian standpoint, for it is necessary in order that the teeth will have the proper grinding and tearing powers, but also from the aspect of the health of the septal tissue, for if the occlusal surface is not properly shaped and the occlusal planes slope toward the proximal surface, the bolus in mastication will have a tendency to be forced in between the teeth and a consequent irritation of the septal tissue will take place, therefore, it is expedient that we carefully reproduce the occlusal markings of the tooth in question and thus make an operation that is not only more useful, but one that is at the same time infinitely more beautiful. In conclusion let me say that I do not by any means use amalgam in all places or for all individuals. If possible I always use gold and prefer not to mix metals in the mouth. I believe that a mouth is in better condition that has only gold restorations and in this day of the gold inlay there is no reason, aside from the expense, that gold can not be used in all restorations, but the poor man has a right to service and a splendid service can be ministered to him with the amalgam filling, and at the same time a technic can be developed that will enable the operator to give the service at a reasonable fee and not rob himself.—*Journal N. D. A.* for July.

